# Regulation of PCNA polyubiquitination in human cells

**DOI:** 10.1186/1756-0500-3-85

**Published:** 2010-03-30

**Authors:** Jan Brun, Roland K Chiu, Bradly G Wouters, Douglas A Gray

**Affiliations:** 1 Ottawa Health Research Institute, Ottawa, ON K1H 8L6, Canada; 2 Department of Biochemistry, Microbiology, and Immunology, University of Ottawa, Ottawa, Ontario, Canada; 3 Apoptosis Research Centre, Children's Hospital of Eastern Ontario, Ottawa, Ontario K1H 8L1, Canada; 4 Department of Health Risk and Toxicology, University of Maastricht, 6200MD Maastricht, the Netherlands; 5 Ontario Cancer Insitute, Princess Margaret Hospital, Toronto, Ontario M5G 2MG, Canada

## Abstract

**Background:**

The ubiquitin-based molecular switch dictating error free versus error prone repair has been conserved throughout eukaryotic evolution. A central component of this switch is the homotrimeric clamp PCNA, which is ubiquitinated in response to genotoxic stress allowing recovery of replication forks blocked at sites of DNA damage. The particulars of PCNA ubiquitination have been elucidated in yeast and to a further extent recently in human cells. However, gaps in the detailed mechanism and regulation of PCNA polyubiquitination still persist in human cells.

**Findings:**

We expand upon several studies and show that PCNA is polyubiquitnated in normal skin fibroblasts, and that this ubiquitination is dependant on RAD18. Furthermore we define the types of DNA damage that induce ubiquitination on PCNA. Cisplatin, methylmethane sulphonate and benzo(a)pyrene-diol-epoxide induce the polyubiquitination of PCNA to the same extent as UV while polyubiquitination is not detected after X-ray treatment. Moreover, we show that ubiquitination of PCNA is not regulated by cell cycle checkpoint kinases ATM-Chk2 or ATR-Chk1. Significantly, we report that PCNA polyubiquitination is negatively regulated by USP1.

**Conclusions:**

Our results demonstrate the importance of PCNA polyubiquitination in human cells and define the key regulator of this ubiquitination.

## Introduction

In recent studies PCNA ubiquitination has been identified as an important modification in human cells [1-4]. Similar to yeast, human PCNA is monoubiquitinated by hRad6/hRad18 (governs error-prone repair) which increases the affinity for translesion polymerase Polη and enables a polymerase switch in response to DNA damage [4]. Moreover hRad18 also interacts with Polη thereby facilitating its localization to sites of DNA damage [5]. PCNA is also polyubiquitinated by the hMms2/hUbc13/SHPRH or hMms2/hUbc13/HLTF complex, which govern error-free repair and disruption of this polyubiquitination leads to genomic instability resulting in increased mutagenesis and gross chromosomal rearrangements [1,2,6,7]. We have previously used cancer cell lines to demonstrate PCNA polyubiquitination. However, the use of cancer cells may result in a distortion of the physiological damage response. Therefore, it remains to be determined whether PCNA polyubiquitination is important in normal human fibroblasts and if this potential modification depends on RAD18.

Another central question is how human cells regulate mono and polyubiquitination of PCNA and hence how they induce or limit the deployment of DNA repair machinery in the presence or absence of damage. One possibility is that PCNA ubiquitination is regulated by cell cycle checkpoint kinases ATR-Chk1 or ATM-Chk2 given their central role in damage surveillance [8-11]. An alternative possibility is deubiquitinating enzymes (DUBs). DUBs are cysteine proteases that cleave ubiquitin from mono and polyubiquitinated substrates. In 2006 Huang et al., were the first to reveal that USP1 negatively regulates monoubiquitination of PCNA in the absence of DNA damage in order to control TLS [12].

In this study we examined the details of PCNA polyubiquitination in normal human fibroblasts and cancer cell lines. Contrary to previous studies, we found that PCNA is mono and polyubiquitinated in primary human skin fibroblasts after UV irradiation and that this modification is dependant on RAD18. In addition, we found that PCNA is polyubiquitinated in response to a variety of DNA damaging agents and that polyubiquitination occurs on K164. Since cell cycle checkpoint kinases are predominantly activated after DNA damage we also sought to determine whether PCNA ubiquitination is regulated by global sensors such as ATR or ATM. Similar to studies in *Xenopus*, *S. pombe *and human cells, we report that mono or polyubiquitination of PCNA is not regulated by the cell cycle checkpoint kinases in human cells [13-15]. Significantly, we confirm that the candidate DUB for negatively regulating PCNA polyubiquitination is USP1.

## Experimental Procedures

### Cell Culture, treatments and transfections

The A549, HEK 293T and HeLa, HCT116, GM038 (skin fibroblasts passage 15) cell lines were cultured in DMEM (Gibco, Invitrogen, Carlsbad, California, United States) supplemented with 10% FBS (Gibco, Invitrogen, Carlsbad, California, United States) and 1× Pen/Strep (Gibco, Invitrogen, Carlsbad, California, United States). UV irradiation (30 J/m^2^) was performed using a UVC germicidal lamp at a fluence rate of 1 J/m^2^/s. Cisplatin (obtained from the Ottawa Hospital Pharmacy, Ottawa, Ontario, Canada) and mitomycin C (Sigma, St. Louis Missouri, United States) were added to cells for 3 hours at a dose of 160 μM and 0.04 μg/ml, respectively, after which they were washed with PBS and supplemented with fresh media. Cisplatin and MMC treated cells were lysed 3 hours post treatment. MMS (Sigma, St. Louis Missouri, United States) was added directly to cells up to 0.02% for 45 minutes followed by immediate lysis. Cells were irradiated with either 4 or 10 Gray of ionizing radiation and harvested 6 hours post irradiation. For caffeine treatment, cells were incubated for 1 hour with either 2.5, 10 or 20 mM caffeine (Sigma, St. Louis Missouri, United States) prior to UV irradiation (30 J/m^2^). GM038, 293T and Hela cells were transfected with siGENOME SMARTpool reagent specific for either human RAD18 or USP1 (Dharmacon Research, Lafayette, Colorodo, United States) using oligofectamine (Invitrogen, Carlsbad, California, United States). The transfections were performed 72 hours prior to harvesting the cells to achieve optimal long-term knockdown. 293T were also transiently transfected with either a GFP-tagged WT PCNA or K164R PCNA DNA plasmid using GeneJuice transfection reagent (Novagen) as per company protocol.

### Immunoblotting

The method and antibodies for immunoblotting have been described elsewhere [1,16].

### Immunoprecipitation

This method has been described elsewhere [1,16].

## Results

### UV irradiation induces PCNA polyubiquitination in normal fibroblasts

**Figure 1:**
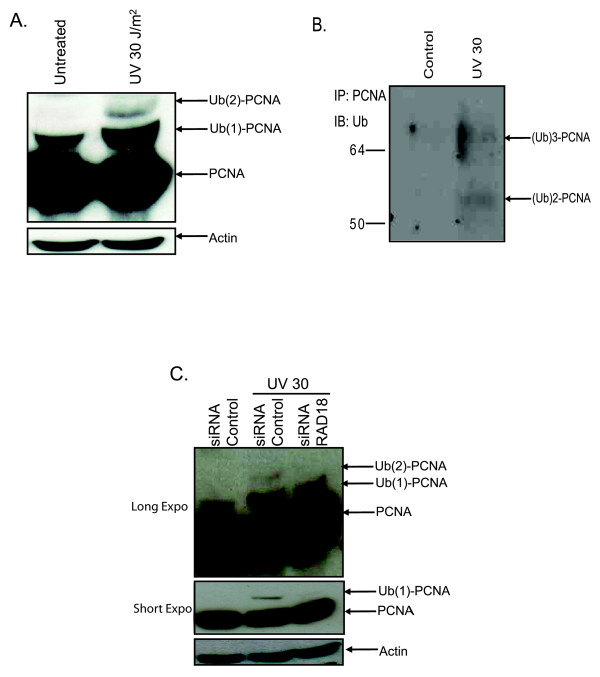
**PCNA ubiquitination in normal skin fibroblasts**. **(A) **GM038 cells were either left untreated or UV irradiated with 30 J/m^2 ^and lysed 3 h post-treatment followed by immunoblotting for PCNA. **(B) **GM038 cells were irradiated with 30 J/m^2 ^UV and lysed in boiling SDS, diluted in lysis buffer and subjected to immunoprecipitation with a PCNA antibody and detected with an anti-ubiquitin antibody. **(C) **GM038 cells were transfected with 100 nM of either control siRNA or siRNA RAD18. 72 hours post-transfection cells were treated as in figure 1A.

To date PCNA polyubiquitination has not been reported in normal human fibroblasts [4,13]. However, in a previous study we were the first to demonstrate that K63-linked polyubiquitination is important in human fibroblasts and that PCNA is both mono and polyubiquitinated in cancer cell lines by Rad18 and Ubc13 [1]. This was further corroborated by Motegi et al. whose studies showed that PCNA polyubiquitination was also dependant on the human RAD5 homolog SHPRH [2]. However, Kannouche et al. only observed monoubiquitination of PCNA in transformed fibroblasts noting that PCNA polyubiquitination was either not important in human cells, occurred at low levels, or quickly turned over [4]. Therefore, we sought to resolve this issue by determining whether PCNA polyubiquitination was physiologically relevant to primary fibroblasts. We chose to investigate primary skin fibroblast GM038 since these cell types are one of the first to encounter environmental mutagens. GM038 cells were irradiated with 30 J/m^2 ^of UVC and 3 hours post-treatment a band representing monoubiquitinated PCNA as well as a second band corresponding to di-ubiquitinated PCNA appeared (Figure 1A). To confirm that these were indeed ubiquitinated forms of PCNA, we immunoprecipitated PCNA followed by immunoblotting with an anti-ubiquitin antibody. Bands corresponding to di and tri-ubiquitinated PCNA were identified in GM038 after UV treatment (Figure 1B). Similar to previous results the anti-ubiquitin antibody failed to reveal monoubiquitinated PCNA [1,16]. This seems to be a property of the antibody since it seems to have a lower affinity for monoubiquitinated substrates.

### PCNA polyubiquitination is dependant on RAD18 in primary fibroblasts

In our previous study we demonstrated that disruption of RAD18 abrogates PCNA polyubiquitination in several cancer cell lines after UV irradiation [1]. Therefore, we sought to determine whether this was the case in human fibroblasts. GM38 cells were targeted with siRNA against RAD18. As expected, RAD18 knockdown resulted in a substantial decrease in the mono and di-ubiquitinated species (Figure 1C). Therefore, PCNA polyubiquitination seems to be an important physiological response to UV damage in healthy cells.

### PCNA is polyubiquitinated in response to a variety DNA damaging agents

**Figure 2:**
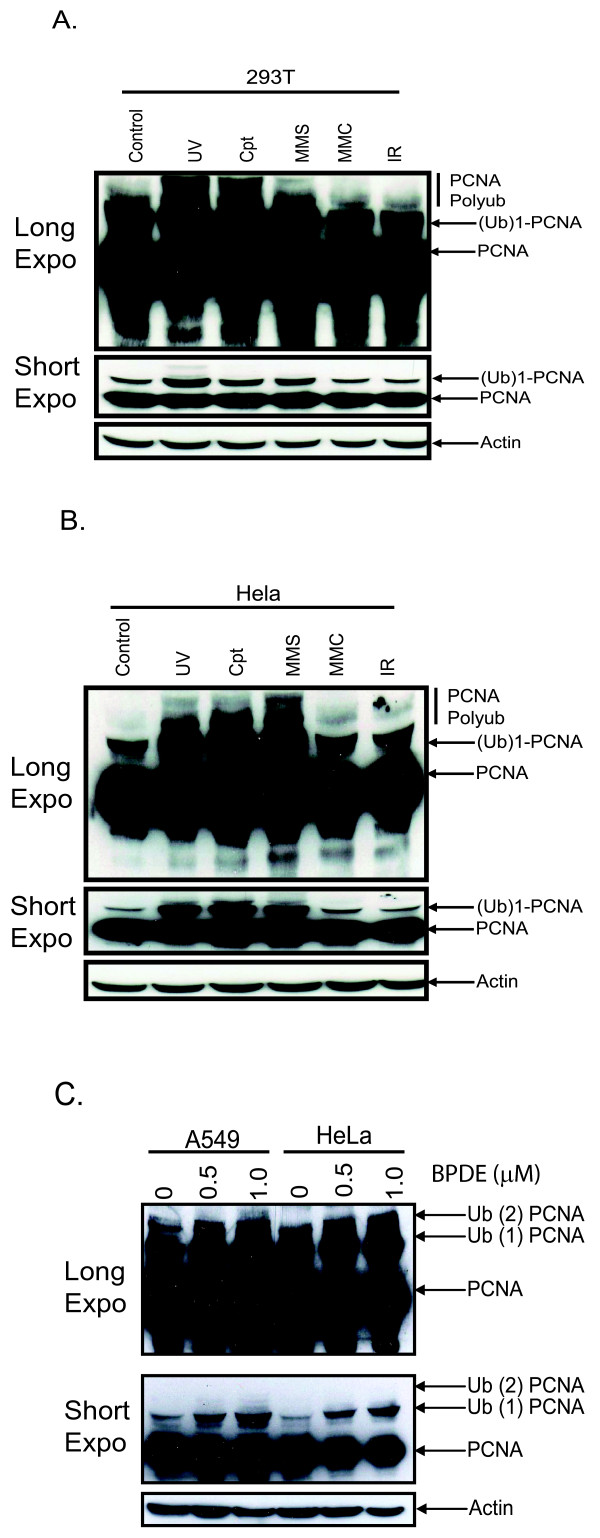
**PCNA is polyubiquitinated in response to a variety of replication stressors**. **(A) **293T cells were untreated or treated with the indicated dose of UV (30 J/m^2^), CPT (160 μM), MMS (0.02%), MMC (40 ng/ml) or ionizing radiation (4 Gy) according to materials and methods. Both longer and shorter exposures are shown. **(B) **HeLa cells were treated the same as in A. **(C) **A549 and HeLa cells were treated with 0, 0.5 or 1 μM BPDE for 1 h and lysed 5 hours post-treatment following by immunoblotting for PCNA.

Thus far, work from our laboratory has shown that UV damage induces PCNA polyubiquitination. We have now examined the ubiquitination status of PCNA after treatment with a variety of DNA damaging agents including the chemotherapeutic drug cisplatin (CPT), environmental carcinogen benzo(a)pyrene-diol-epoxide (BPDE), methylmethane sulfonate (MMS), mitomycin C (MMC) and X-irradiation (X-ray). We were able to detect mono and polyubiquitinated PCNA in 293T, and HeLa cells after CPT, and MMS treatment to approximately the same extent as that of mono and polyubiquitination after UV irradiation (Figure 2A-B). We were also able to detect a dose dependant increase in mono and polyubiquitination in HeLa and A549 after BPDE treatment (Figure 2C). Moreover, this ubiquitination occurred in cells expressing WT K164 PCNA but not in cells expressing a mutant K164R PCNA, which indicates that this is the major site on which PCNA is ubiquitinated in human cells [Additional file 1]. Finally we were unable to detect an increase in mono or polyubiquitinated PCNA after treatment with MMC and X-ray which may indicate that PCNA ubiquitination is a specific response to certain types of DNA damage or occurs with different kinetics based on the type of DNA damage.

### PCNA polyubiquitination is regulated by USP1 and not by cell cycle checkpoint kinases

**Figure 3:**
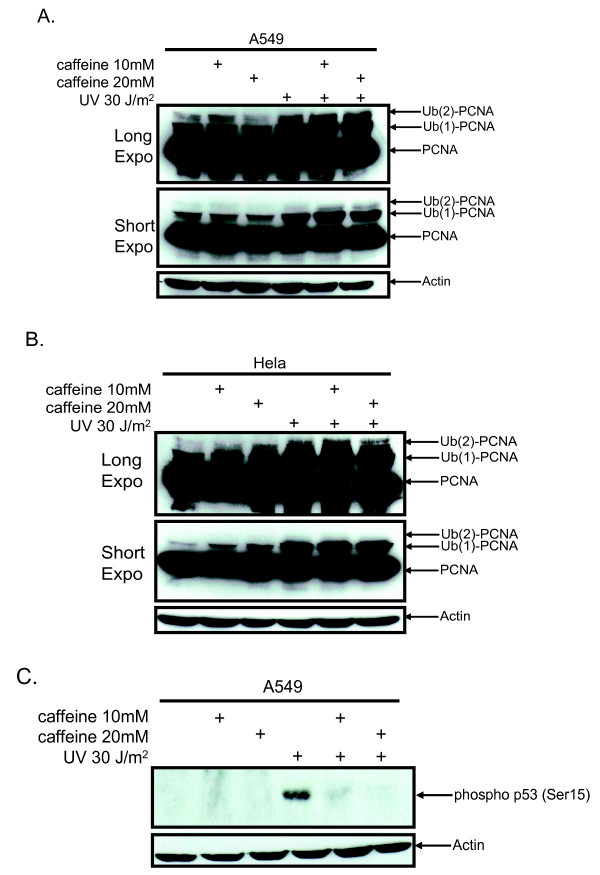
**Inhibition of ATR or ATM does not abrogate PCNA ubiquitination**. **(A) **A549 and **(B) **HeLa cells were incubated with the indicated doses of caffeine 1 h prior to UV irradiation. Six hours after irradiation cells were lysed followed by immunoblotting for PCNA. **(C) **A549 cells were treated as in A followed by immunoblotting for phosphorylated S15 on p53.

ATM and ATR are cell cycle checkpoint kinases activated in response to DNA damage [17,18]. To determine whether ATR or ATM regulate PCNA ubiquitination we analyzed the effect of inhibiting their kinase activity by incubating A549 and HeLa cells with 10 or 20 mM caffeine prior to UV irradiation. We demonstrate that PCNA is mono and polyubiquitinated to the same extent in the presence or the absence of caffeine (Figure 3A and 3B). It is interesting to note that after UV irradiation there is a modest increase in mono and polyubiquitination in the caffeine treated A549 cells (Figure 3A, short exposure) while levels of PCNA mono and polyubiquitination are similar in untreated or caffeine treated HeLa cells (Figure 3B). To confirm that our caffeine doses were inhibiting ATR or ATM we analyzed the p53 phosphorylation status at serine 15 after UV irradiation in the A549 cell lines, which express wild type p53 [19]. It was observed that 10 or 20 mM caffeine abolished the phosphorylation of p53 while a strong phosphorylated ser15 p53 signal was present in the non-caffeine treated but UV irradiated A549 cells (Figure 3C).

**Figure 4:**
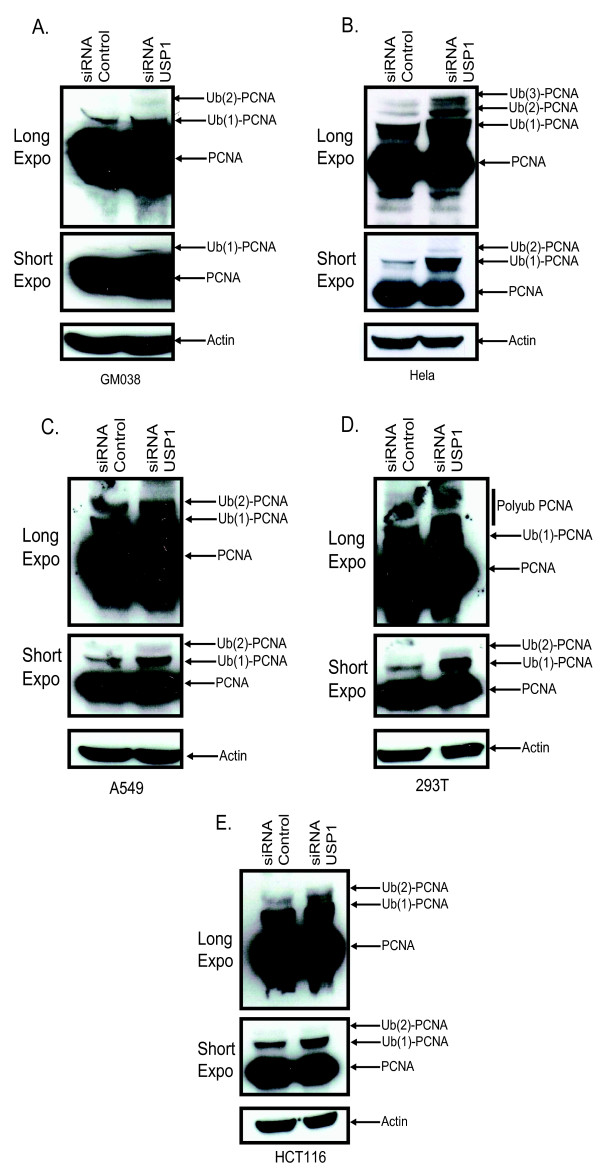
**Knockdown of USP1 leads to increase PCNA ubiquitination**. **(A-E) **All indicated cell lines were transfected with 100 nM of either control siRNA or siRNA USP1. 72 hours post-transfection cells were lysed and immunoblotted for PCNA.

A recent study implicating USP1 in negatively regulating monoubiquitinated PCNA led us to evaluate whether USP1 is also involved in regulating PCNA polyubiquitination [12]. Using siRNAs targeting USP1 in human cells, we observed a substantial increase in the levels of both monoubiquitinated and polyubiquitinated PCNA in GM038, HeLa, A549, 293T, and HCT116 cells (Figure 4A-E). This adds to the accumulating evidence that suggests USP1 as a regulator of PCNA polyubiquitination.

## Discussion

In a previous study we were the first to show that K63-linked polyubiquitination is important in skin fibroblasts but did not demonstrate the substrate involved in these cells [1]. Here we expand upon that study and demonstrate that PCNA is the substrate for K63-linked polyubiquitination and that PCNA polyubiquitination is a normal physiological response to DNA damage in normal diploid skin fibroblast and that this process is dependant on RAD18. Moreover, we report that PCNA is polyubiquitinated in response to a variety of genotoxic agents that distort DNA including UV, CPT, MMS and BPDE. This would suggest that PCNA polyubiquitination is a common mechanism used to protect cells against the mutagenic effects of DNA damage.

Thus far our data have shown that PCNA polyubiquitination is induced by several DNA damaging agents in a variety of human cell lines. Interestingly, the same replication stressors used in this study also activate a global checkpoint response which is governed by two serine/threonine kinases ATM and ATR. Their activation facilitates the recruitment and phosphorylation of other proteins, including Chk1 and p53, which consequently arrest cells at G1 [20,21]. Our interest in this pathway in regulating PCNA ubiquitination stems from studies which demonstrated that ATM and ATR activation are necessary for phosphorylation and subsequent ubiquitination of FancD2 [18]. Since FancD2 colocalizes with PCNA after DNA damage we postulated that ATR or ATM may similarly regulate PCNA ubiquitination. Inhibition of ATR or ATM with caffeine showed no disruption in PCNA ubiquitination. In fact it showed a marginal increase in both PCNA mono and polyubiquitination, at least in A549 cells. These results were similar to those recently reported by Chang et al., and Niimi et al [14,15]. However, this does not rule out that PCNA ubiquitination is regulated by cell cycle checkpoint kinases; rather, it may point to alternative pathways that may compensate for the inhibition of ATM and ATR. Recently, the p38 SAPK-MK2 pathway was demonstrated to be involved in the response to DNA damage converging on similar substrates activated by ATR and ATM [9]. Since caffeine has been shown to upregulate p38 activity one could postulate that the p38 pathway is compensating for the loss of ATR/ATM [22]. Future studies will be required to determine whether inhibition of ATR, ATM and the p38 pathway in combination abrogates PCNA ubiquitination.

Recent studies point towards DUBs as another means to regulate PCNA ubiquitination [6]. For example, USP1 has been implicated in the constitutive deubiquitination of monoubiquitinated PCNA and USP1 functions independently of the cell cycle checkpoint kinases similar to PCNA polyubiquitination [12,15]. Here we show that USP1 is also involved in deubiquitinating polyubiquitinated PCNA, the implications of which are unknown. Functionally, Huang et al. showed that disruption of USP1 lead to increased spontaneous and UV induced mutagenesis [12]. They postulated that the increase in mutagenesis could be due to the dysregulated function of Polη or recruitment of other error-prone polymerases to the damaged sites [12]. In light of our data showing an increase in PCNA polyubiquitination after USP1 disruption, another explanation for the increased mutagenesis could be the contribution of unscheduled, illegitimate and dysregulated homologous recombination. During the writing of this manuscript Motegi et al, also published corroborating data demonstrating an increase in polyubiquitination of PCNA after abrogating USP1 expression [6].

In conclusion, our data show that PCNA polyubiquitination is an important response to DNA damage and that it is constitutively regulated by USP1 and not by cell cycle checkpoint kinases. A challenge for future investigations will be to elucidate whether PCNA ubiquitination is a compartmentalized response simply regulated by USP1 alone or regulated by other signaling pathways.

## Abbreviations

PCNA: proliferating cell nuclear antigen; UV: ultra-violet; CPT: cisplatin; MMC: mitomycin C; MMS: methylmethane sulphonate; USP: ubiquitin specific protease; BPDE: benzo(a) pyrene diol-epoxide; DDT: DNA damage tolerance; TLS: translesion synthesis; K63: lysine 63.

## Competing interests

The authors declare that they have no competing interests.

## Authors' contributions

The contents of this manuscript were written by JB and edited by DAG, RKC, BGW. JB performed the experiments. JB, RKC, BGW and DAG analyzed the data. JB and RKC provided necessary the analysis tools reagents and materials. All authors have read and approved the final manuscript.

## Supplementary Material

Additional file 1 **Polyubiquitination of PCNA occurs at K164**. In yeast, PCNA is mono and polyubiquitinated at K164 [23]. Recent evidence demonstrate that PCNA polyubiquitination catalyzed by human enzymes occurs on K164 *in vitro *[3]. To verify that K164 on PCNA is the target for polyubiquitination in human cells **(A) **293T cells were transiently transfected with a GFP tagged wild type (WT) or mutant K164R PCNA plasmid. Post-transfection (48 h) cells were UV irradiated, lysed and immunoblotted for PCNA. This system distinguishes between endogenous and recombinant modified PCNA. Thus we are able to clearly observe the effect of the mutation K164R mutation by Western blot analysis. GFP-PCNA, GFP-mono and di-ubiquitinated forms of PCNA were detected in the cells transfected with WT PCNA, however, they were notably reduced in cells transfected with the K164R mutant. Click here for file
