# Supplementary figures and images for: Regulation of PCNA polyubiquitination in human cells
Source: BMC Res Notes. 2010 Mar 30;3:85. doi: 10.1186/1756-0500-3-85 (PMC2867771; doi:10.1186/1756-0500-3-85)

293T

WT PCNA  
PCNAK164R  
UV 30 J/m<sup>2</sup>

+

+

+

+

+

+

+

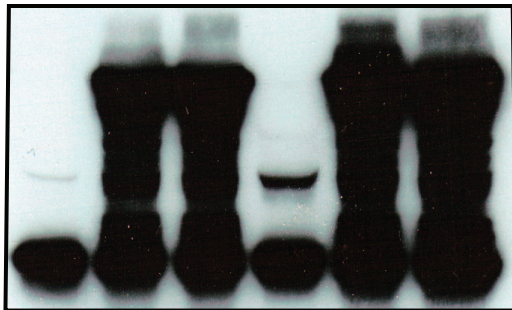

← (Ub)<sub>2</sub>-PCNA-GFP

← (Ub)<sub>1</sub>-PCNA-GFP

← GFP PCNA

← (Ub)<sub>1</sub>-PCNA

← PCNA

Supplement: Additional file 1 — Polyubiquitination of PCNA occurs at K164. In yeast, PCNA is mono and polyubiquitinated at K164 [23]. Recent evidence demonstrate that PCNA polyubiquitination catalyzed by human enzymes occurs on K164 in vitro [3]. To verify that K164 on PCNA is the target for polyubiquitination in human cells (A) 293T cells were transiently transfected with a GFP tagged wild type (WT) or mutant K164R PCNA plasmid. Post-transfection (48 h) cells were UV irradiated, lysed and immunoblotted for PCNA. This system distinguishes between endogenous and recombinant modified PCNA. Thus we are able to clearly observe the effect of the mutation K164R mutation by Western blot analysis. GFP-PCNA, GFP-mono and di-ubiquitinated forms of PCNA were detected in the cells transfected with WT PCNA, however, they were notably reduced in cells transfected with the K164R mutant. [file 1756-0500-3-85-S1.PDF]
